# Eco-Sattva: a Buddhist response to the plastic waste crisis in Thailand

**DOI:** 10.3389/fpsyg.2025.1482883

**Published:** 2025-01-27

**Authors:** Yaoping Liu, Pharatt Run, Metteyya Beliatte

**Affiliations:** Institute of Science Innovation and Culture, Rajamangala University of Technology Krungthep, Bangkok, Thailand

**Keywords:** Eco-Sattva, Wat Chak Daeng, monk’s robe, Thailand, plastic waste crisis

## Abstract

The plastic waste crisis in Thailand has emerged as a substantial environmental dilemma, significantly exacerbating pollution in both marine and terrestrial ecosystems. Although previous study has mostly focused on technological and policy solutions, it is still necessary to conduct a thorough evaluation of the approach to this issue that is driven by cultural and spiritual values. This study aims to examine the influence of eco-sattva principles on the management of plastic garbage and the Buddhist communities in Thailand. It also seeks to assess the effects of Eco-Sattva’s teachings by conducting a case study at Wat Chak Daeng. A comprehensive series of interviews were carried out with a total of 15 participants, encompassing priests, community leaders, and volunteers. The transcribed data was processed and examined using the NVivo program. The interview findings demonstrate that the lessons of Eco-Sattva on plastic waste management are extremely advantageous in terms of their practical use and execution. This study investigates and analyses the three-step waste management process at Wat Chak Daeng for plastic waste management, which involves reducing, reusing, and recycling plastic trash. This study provides a significant contribution by showing that the resolution to the plastic waste crisis is not solely reliant on technical or policy-focused approaches, but may also be derived from spiritual principles.

## Introduction

The Eco-Sattva concept has arisen as a Buddhist solution to environmental concerns, such as the plastic trash epidemic in Thailand, in recent decades (Komin, 2019; Javanaud, 2020; Speece, 2023). Eco-Sattva is a compound term derived from the words “Eco,” about the study of ecology or the natural environment, and “Sattva,” a concept rooted in Buddhism that signifies the commitment of sentient beings or enlightened individuals to the preservation and well-being of all living organisms. This concept is based on Buddhist teachings of universal and non-violent compassion (Ahimsa), which promotes protecting the environment and all living beings from harm. Eco-Sattva contributes to raising environmental consciousness through tangible efforts guided by Buddhist principles (Edelglass, 2021; Tormen, 2023), including simplicity, mindfulness, and shared accountability within the framework of the plastic trash epidemic.

Various studies, including those conducted by Loy (2003), Kaza (2008), Harris (2012), Schmithausen (2019), and Grunwald (2023), have investigated the role of religion in addressing environmental concerns. Loy (2003) underscored the importance of spirituality in advancing the environmental movement, while Kaza (2008) demonstrated that meditation can be used to improve environmental awareness. Furthermore, Harris (2012) examined the contribution of Buddhist organizations to environmental conservation efforts in Southeast Asia. Gross (2014) examined the idea of Buddhism as a moral basis for conserving the environment. According to Schmithausen’s (2019) research, Buddhism significantly contributes to alleviating environmental harm by following the principle of Ahimsa. Grunwald’s (2023) research introduces the notion of Ecosattvas and ecodharma as a contemporary strategy for ecological preservation, which serves as the foundation for this study. While these studies have made notable contributions, there needs to be research that particularly examines the impact of Eco-sattva practices on reducing plastic waste, nor the strategies employed by Buddhist communities in Thailand to address the issue of plastic trash.

Wat Chak Daeng is a Buddhist temple in the Bang Krachao Area of the Samut Prakan province in Thailand. It is well-known for its long-standing sustainability programs that have been in place for over 20 years. The temple has demonstrated the practical application of Eco-sattva teachings in addressing environmental issues. The temple has successfully transformed plastic waste into robes for the monks using basic technology. Therefore, this phenomenon necessitates further investigation, as evidenced by this investigation. The objective of this investigation is to undertake a comprehensive analysis of the Eco-sattva concept’s implementation in the context of the plastic waste crisis in Thailand. The case study conducted at Wat Chak Daeng will be the primary focus of evaluating the Buddhist community’s role in implementing sustainable solutions for the management of plastic waste.

## Literature review

### Theoretical background

Eco-Sattva (written as “Ecosattva” as well), a neologism combining ecology and bodhisattva, refers to an enlightened person who helps sentient beings and the environment (Grunwald, 2023). The term “Eco-Sattva” has been introduced to denote bodhisattvas who are actively involved in environmental activism (McAnally, 2018). As explained by Loy (2019) Eco-Sattva integrates bodhisattva consciousness acquired through meditation with environmentally-focused campaigning inspired by Buddha’s teachings. Eco-Sattva refers to spiritual actions that safeguard nature and foster environmental peace. The Eco-Sattva, inspired by the bodhisattva, possesses both meditative inner insight of ultimate truth and outward compassion for soil, the Earth, and all living entities (Grunwald, 2023). Western Buddhist practitioners, particularly Zen and Tibetan adherents, along with socially engaged activists, have developed the concepts of Eco-Sattva, Eco-Sangha, and Eco-Dharma through innovative endeavors, which have subsequently impacted the Asian Buddhist community (Kaza, 2003). The Eco-sattva concept, informed by relevant Buddhist teachings, such as the practice of detachment from the ego, the significant relationship between intention and the karmic consequences of actions, as well as the principles of loving-kindness, non-harming, compassion, and interdependence, has become an integral part of Buddhist environmentalism. McAnally (2018) points out that this phrase was first introduced in the 1990s to designate a collective of Zen practitioners at Green Gulch Farm in Sausalito, California. When implemented, the activists are mandated to adhere to follow the “Eco-Sattva Vows” to promote the health, vitality, and well-being of all beings, visible and invisible, near and far, born and yet-to-be-born. While both are driven by environmental concerns, Buddhist environmentalism differs from other forms of environmental activism and eco-religious movements due to its unique foundation in Buddhist thought and practice (Kaza, 2003). This concept, inspired by Buddhism, can be utilized by any Buddhist individual or organization to enhance the environment. This notion, rooted in contemporary ecological theories and environmentalism, is further enriched by Buddhist thinking and in return contributes to ecological theory and environmental practice by providing additional philosophical perspective.

### Plastics’ classification

**Figure 1:**
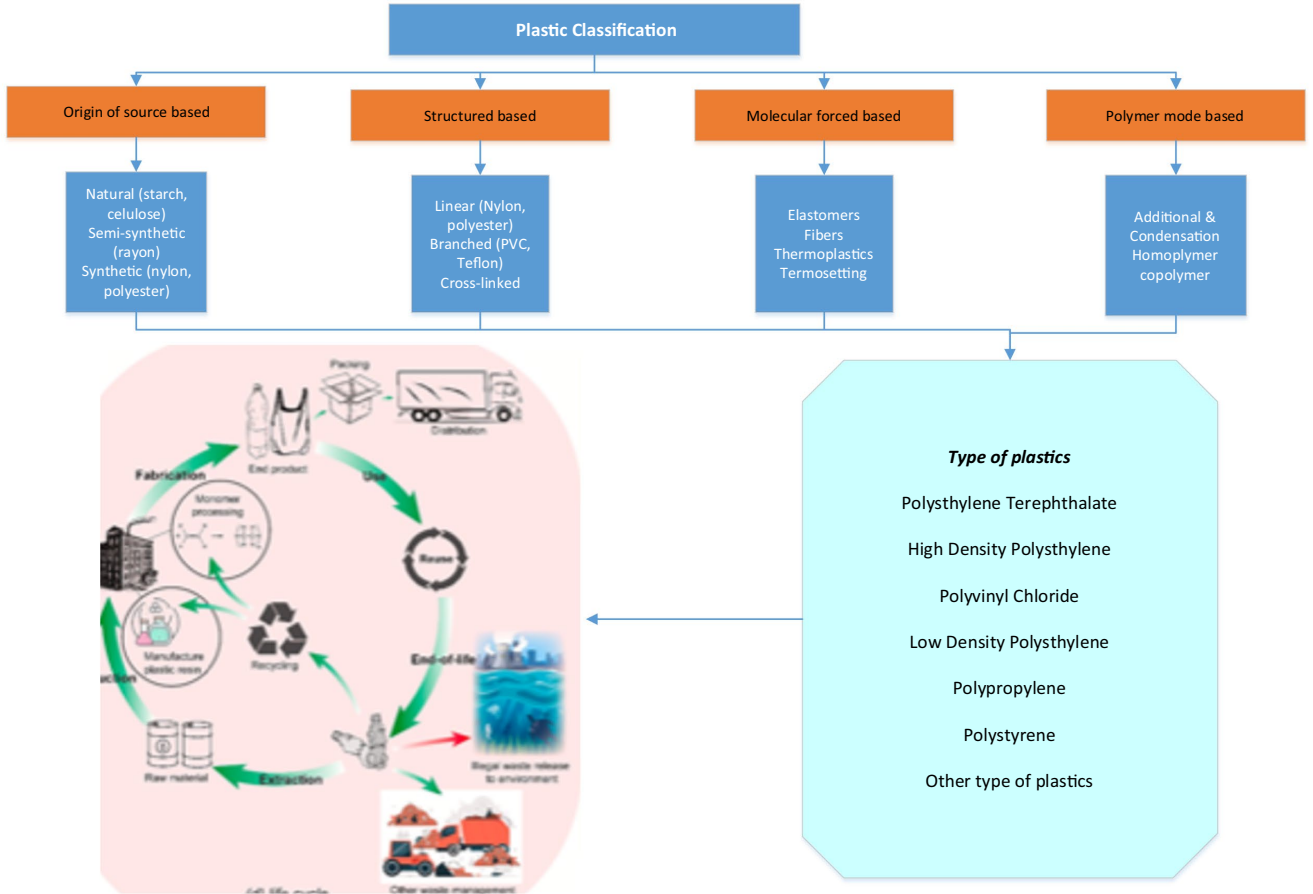
Plastic classification.

Plastics can be categorized according to many criteria, including their source, structure, molecular factors, polymerization mechanism, dependent on temperature properties, and preservation procedures. The classification of plastics depends on their origin and can be divided into three subcategories: natural, partially synthetic, and synthetic. Natural polymers have existed since prehistoric times. Natural plastics, including lignin, chitin, and starch, are polymers derived from organic materials like horn, amber, and tortoise. Synthetic polymers, such as silicone, polyethylene, polystyrene, and polyester, are produced from petrochemicals (Geyer, 2020; Nanda and Berruti, 2021). Semi-synthetic plastics refer to natural polymer compounds, such as cellulose-based polymers, that have undergone chemical modifications (Wang et al., 2021). The categorization based on structure can be classified into linear, branching, or cross-linked. Furthermore, molecular-based categorization can be categorized into four distinct types: elastic materials, fiber, thermoplastic, and plastics that thermoset (Figure 1).

Alternative techniques can also be utilized for the specific categorization of plastics. For instance, the categorization that relies on the physio-chemical characteristics and uses of plastics mainly concentrates on thermoplastics and thermoplastic. Figure 1 illustrates the precise categorization of plastics according to their physio-chemical properties, uses, material origin, biodegradable life cycle, and adherence to international criteria. Plastics might be classified into two categories, thermoplastic and thermoplastic, based on their physiochemical qualities before and after heat treatment. Thermoplastics can undergo a phase change from solid to liquid when heated, allowing them to be molded into various shapes. They then return to a solid state when cooled. Thermoplastics have the ability to undergo repetitive deformation with the characteristic property of plasticity. According to Al-Salem et al. (2009), thermoplastics account for 80% of the overall plastic use. Thermosetting plastics undergo immediate decomposition when subjected to continuous heating rather than undergoing deformation (Xie et al., 2024). Plastics can be categorized into general, engineering, and functional types based on their applications. Plastics generally exhibit shared characteristics that make them suitable for a broad spectrum of uses. Engineering plastic exhibits exceptional comprehensive characteristics, including high stiffness, hardness, and temperature resistance. As a result, engineering plastics are utilized as construction materials to substitute metal. Functional plastic possesses unique properties such as electrical conductivity, defense against radiation, resilience to corrosion, and permeability to magnets (Sheha, 2024).

Plastics can be categorized into four divisions based on their material origin and biodegradability features. The primary distinction between non-biodegradability and biodegradability lies in their capacity to decompose in the natural environment. Biodegradable plastics undergo natural decomposition, while non-biodegradable polymers remain unchanged over an extended duration (Cakmak, 2024). Plastics can be categorized depending on their life cycle into single-use plastic, the primary cause of heavy pollution, and reusable plastic (Liu et al., 2021). In addition to the aforementioned conventional categorizations, the Society of the Plastic Industry (SPI) implemented a numerical coding system for plastics (Figure 1). The classification assigns numbers 1–7 to various kinds of materials in the following sequence: 1 for the plastic polyethylene terephthalate, 2 for polyethylene with a high density (HDPE), 3 for vinyl chloride, 4 for low-density polyethylene, 5 for polypropylene, 6 for polystyrene, and 7 for other types of plastics (Figure 1). This categorization aids customers and recyclers in streamlining the recycling procedure. Plastics can be classified as either bio-based or fossil-based compounds. Bioplastics can be categorized into different groups based on their breakdown properties: biodegradable (derived from natural sources), biodegradable (derived from petrochemicals), and non-biodegradable (derived from natural sources).

### Plastic as a waste material

Since most plastic waste is resistant to natural breakdown, the problem of plastic trash has grown considerably more prevalent. Inadequate regulations on creating, using, and disposing of plastic trash are standard in poor countries, and many of these countries also need more advanced technological infrastructure (Chisholm et al., 2021). The United Nations has issued a declaration acknowledging that plastic pollution is a significant environmental concern for land and sea ecosystems (Phelan et al., 2022; Islam et al., 2024). The conversion of plastic from a functional ‘item’ to a troubling ‘byproduct’ has emerged as a popular subject worldwide. The global production of plastic trash is steadily rising due to three primary factors: (i) The capacity of plastic to substitute conventional materials like ceramics, wood, and glass, (ii) the rise of the population, and (iii) the convenience availability of consumer society. Most individuals relocate from rural regions to developed areas, significantly increasing the urban population from 751 million to 4.2 billion. This group is characterized by its convenient access to consumer society (Chhetri, 2023).

There is a significant global production of plastic waste due to poor management, inefficient waste management methods, and ongoing disposal (Tejaswini et al., 2022). According to Geyer et al. (2017) and Geyer (2020), over 5,800 million metric tons (Mt) of plastic garbage that needed to be correctly managed were released into the environment by the end of 2015. Plastics persist in the environment for extended periods, often lasting for millennia, because of their stability, endurance, and resistance to biodegradation. This is primarily due to their considerable molecular weight, complex three-dimensional structure, and hydrophobic properties (Fang et al., 2015; Zhang et al., 2022). Plastics experience several weathering mechanisms in the environment, including photodegradation, thermal oxidation, hydrolysis, biodegradation, and fragmentation (Wang et al., 2022). Plastics are found in several environments, including deserts, farms, mountains, and oceans, as documented by Citterich et al. (2023).

This situation is also evident in both urban and rural areas of Thailand, where the management of plastic trash could be operating more efficiently. Additional evidence from Jambeck et al. (2015) confirms that Thailand is one of the leading countries in terms of annual plastic pollution in the ocean. This is further exacerbated by the absence of waste management infrastructure and public foreness regarding the significance of reducing, recycling, and repurposing plastic waste. In contrast, local initiatives, such as Wat Chak Daeng, are beginning to demonstrate the potential to resolve this issue through Eco-Sattva, a spiritual teaching and community-based approach. This instruction promotes the direct responsibility of individuals for waste management, which encompasses reducing plastic consumption, recycling, and repurposing. This is consistent with the 3R principle (Reduce, Reuse, and Recycle), which is a significant global concern in the context of the plastic waste crisis (Geyer et al., 2017; Wang et al., 2020).

Although initiatives like Wat Chak Daeng have been made, implementing spiritual values-based approaches must be improved in numerous locations. Huang et al. (2022) found that a significant challenge in addressing plastic trash in underdeveloped nations is the need for more technology utilization and the absence of cooperation between local efforts and national policy. Hence, this research aims to explore the use of Eco-Sattva teachings in plastic waste management and examine how its principles might be integrated with contemporary approaches to effectively tackle the ecological crisis sustainably.

### Waste management

Speece (2019) discussed the application of Buddhist principles to improve waste management and promote sustainable development in the field of economics. Song proposed that implementing recycling systems and adopting a sufficiency economy philosophy are practical approaches for trash management. According to Manomaivibool et al. (2018), a “sufficiency economy” approach can be employed for the purpose of zero-waste management, explicitly focussing on reducing, reusing, and recycling, benefitting all stakeholders. According to Rukspollmuang (2022), the three fundamental principles of the “sufficiency economy” play a crucial role in promoting waste management. The first notion of “moderation” centers around prudent and conscientious consumption, refraining from an excessive craving for material items that may lead to extra waste. Another notion of “reasonableness” highlights the importance of undertaking logical and compassionate assessments of actions and consequences, forbidding any negligent acts of discarding goods, and advocating for appropriate waste disposal. The additional idea of “self-immunity” pertains to the capacity to deal with challenging situations by implementing responsibility in managing trash at the home level, hence decreasing reliance on others.

The tagline “3R waste for the heart” embodies the notion of a sufficiency economy, promoting a zero-waste approach through reducing, reusing, and recycling. Manomaivibool et al. (2018) and Sarkar (2022) combined waste disposal and sufficiency economy principles with the zero-waste city concept rooted in Buddhist economics. Collaboration with Buddhist temples and private-public entities, mainly in garbage disposal, is an urgent aspect of waste management. Traditionally, the established concepts in waste management at the temples have been widely embraced by the community and via joint efforts from both private and public sectors. Ahmed and Ali (2006) emphasized that waste management includes the public’s involvement, privatization, citizens’ rights, accountability, transparency, and financial efficiency. Batista et al. (2021) categorized waste management with a specific emphasis on creating guidelines and implementing waste management actions.

This paper presents a waste management system consisting of three primary steps: reduction, reuse, and recycling. Reducing waste at its source helps minimize the load and cost of transporting and disposing of garbage. Reuse involves the use of reusable things and reworking objects instead of discarding them. Recycling is the act of segregating materials that can be recycled from those that cannot and depositing them at a designated facility for the recycling process. Prior research has primarily concentrated on collecting and segregating rubbish within the temples (Rajendran et al., 2019). Muhammad et al. (2017) and Tiew et al. (2019) proposed a correlation between waste management procedures and religious observances. Religious activities can contribute to the availability of localized resources for reusing, reducing, and recycling, as Mohamad et al. described. Xiao et al. (2017) studied waste management, specifically examining the effectiveness of involvement, data administration, and trash distribution. Their research suggests trash disposal could promote collaboration, collecting tactics, and motivation.

Only PET bottles that have been cleaned and compressed into blocks by volunteers are suitable for recycling. They are brought to a factory, where one machine makes polyester threads and another shreds the plastic. Before being dyed saffron, the threads are mixed with cotton and antimicrobial strands to form a cloth. Although monks were never formally trained in science, the accumulation of plastic debris along the riverbank prompted his interest in waste management and, eventually, the concept for a recycled robe. The first pair of “recycled robes” was released in 2018 following 3 years of development and cooperation with a chemical firm. The cloth that is produced is silky and smooth rather than as harsh as plastic bags. At least 40 tons of plastic waste have already been saved every year from Wat Chak Daeng waste management. Waste management of Wat Chak Daeng is not only PET but also includes aluminum cans, plastic bags, beverage cartons, glass bottles, styrofoam, paper, and paper cartons (From observation and interviews).

### Research on Buddhist response to the plastic waste crisis in Thailand

Academic interest in the environmental catastrophe has focused on studying religious responses, notably Buddhism. Preliminary research primarily examined the correlation between Buddhist moral values and environmental concerns in a broad sense. An illustration of this is the study conducted by Darlington (2012), which examined the potential application of Buddhist principles, such as karma and interdependence, in bolstering environmental advocacy. In contrast, Kaza (2008) examined how meditation and mindfulness techniques can increase the awareness of the ecological implications of individuals’ daily activities. This research was instrumental in developing concepts such as Eco-sattva, which combines spiritual principles with environmental accountability.

However, there needs to be more studies mainly focussing on the plastic trash crisis in Thailand, despite attempts to link Buddhist teachings with environmental issues. Harris (2012) conducted a study that focused on the environmental conservation initiatives of Buddhist communities in Southeast Asian nations, such as Thailand. The study analyzed how these groups used spiritual customs and rituals to preserve the environment. While this research is pertinent, its primary focus lies on conserving forests and other natural resources, with only briefly mentioning the problem of plastic trash. This indicates that the Buddhist viewpoint has yet to be fully implemented in addressing the issue of the plastic waste crisis, namely in Thailand.

In addition, Gross (2014) showed that the Buddhist concept of non-violence, known as Ahimsa, can provide a solid basis for environmental ethics. Gross argues that Buddhist communities can significantly influence advocating for adopting more ecologically conscious lifestyles. However, Gross needs to give more importance to the problem of plastic pollution, especially in Thailand, as shown by a previous study. This suggests that a scarcity of material establishes a connection between Buddhist teachings and practical measures to address the plastic waste challenge. Recent scholarly investigations, such as the study undertaken by Schmithausen (2019), have begun establishing a correlation between Buddhism and more precise ecological difficulties. For the most part, however, this research concentrates on theoretical factors, and it is possible that they do not include any practical examples from actual life circumstances in Thailand. Schmithausen recognized the capacity of Buddhist teachings to promote environmentally conscious conduct. Still, there needs to be more research on their practical implementation, particularly regarding handling plastic garbage in Buddhist nations like Thailand.

Research Question:

1. How can the principles of Eco-Sattva be implemented in managing plastic waste to address the plastic waste situation in Thailand effectively?
2. How do Eco-Sattva’s teachings affect trash management and Buddhist communities in Thailand?

## Methods

### Case study design

The design of our case study was qualitative and relied on conducting in-depth interviews. The utilization of the case study approach is a crucial methodology for addressing research enquiries, particularly in the context of under-explored subjects like Eco-sattva and the plastic waste crisis in Thailand. According to Baskarada (2014), a qualitative case study approach seeks to achieve a comprehensive grasp of intricate insights, allowing researchers to investigate the occurrences in depth. We selected this design because of its capacity to incorporate the diverse and inclusive nature of the Eco-sattva philosophy. This design is employed as a comprehensive evaluation method to rigorously analyze data and guarantee the quality of a study report.

### Case setting

The case study occurred at Wat Chak Daeng, a temple that has emerged as a paradigm for plastic waste management initiatives in Thailand. The temple is situated in a region dedicated to advancing a low-carbon metropolis, which entails implementing several techniques to diminish carbon emissions, including trash management. The waste management practices of Wat Chak Daeng include the segregation of garbage at its origin, minimizing the utilization of plastic materials, implementing a well-organized waste collection system, and providing storage facilities that facilitate recycling. This method helps decrease plastic trash and assists broader initiatives to reduce the city’s carbon footprint.

The background of this case is important and relevant due to the little research conducted on waste management practices in Thai temples, especially within the framework of a low-carbon city. Prior research has predominantly emphasized waste management’s technical and policy dimensions, neglecting to examine the involvement of religious institutions and local communities in these endeavors. Wat Chak Daeng exemplifies integrating spiritual and religious principles with practical environmental initiatives by upcycling plastic trash into monks’ robes.

### Participant

This study employed purposive sampling to choose instances for the investigation of plastic waste management techniques influenced by the idea of Eco-Sattva. The sampling approach is to choose representative subjects that accurately reflect the topic being studied (Robinson, 2014; Palinkas et al., 2015). Initially, a compilation of prospective samples was generated. Wat Chak Daeng is mainly dedicated to managing plastic garbage, with a significant community focused on this task. This community comprises monks, volunteers, and local community members who collaborate in several activities related to trash management. They engage in garbage segregation, recycling, and plastic waste conversion into valuable commodities, such as monk robes. Managing waste using the 3Rs is regarded as a low-carbon endeavor in waste management. Next, we reached out to influential individuals actively engaged in garbage management at Wat Chak Daeng. We acquired consent from a total of 15 people. Table 1 displays the three occupational categories of primary informants and their respective responsibilities in managing waste.

**Table 1:** The participants for the interview.

No	Occupation	Age (years)	Role of the participants in the process
1	Monk 1	30–35	Initiator, guidance, supervision, leading by involving in the activities by both advising and rendering labor.
2	Monk 2	40–45	Initiator, guidance, supervision, leading by involving in the activities by both advising and rendering labor.
3	Monk 3	25–30	Initiator, guidance, supervision, leading by involving in the activities by both advising and rendering labor.
4	Monk 4	50–55	Initiator, guidance, supervision, leading by involving in the activities by both advising and rendering labor.
5	Monk 5	40–45	Initiator, guidance, supervision, leading by involving in the activities by both advising and rendering labor.
6	Monk 6	35–40	Initiator, guidance, supervision, leading by involving in the activities by both advising and rendering labor.
7	Monk 7	45–50	Initiator, guidance, supervision, leading by involving in the activities by both advising and rendering labor.
8	Monk 8	55–60	Initiator, guidance, supervision, leading by involving in the activities by both advising and rendering labor.
9	Community leader 1	50–55	Collaborative performer, persuasion of participants, actively engaging by finding volunteers and providing occasional labor support.
10	Community leader 2	55–60	Collaborative performer, persuasion of participants, actively engaging by finding volunteers and providing both advise and occasional labor support.
11	Community leader 3	45–50	Collaborative performer, persuasion of participants, actively engaging by finding volunteers and providing both advice and occasional labor support.
12	Community leader 4	50–55	Collaborative performer, persuasion of participants, actively engaging by finding volunteers and providing both advise occasional labor support.
13	Volunteer 1	35–40	Collector, receiving instruction, following guidelines provided by the monks and the community leaders, actively rendering labor.
14	Volunteer 2	40–45	Collector, receiving instruction, following guidelines provided by the monks and the community leaders, actively rendering labor.
15	Volunteer 3	45–50	Collector, receiving instruction, following guidelines provided by the monks and the community leaders, actively rendering labor.

### Case collection

Participants were interviewed extensively to explore their approach to integrating Buddhist environmental principles into waste management practices. The research project team created interview rules by utilizing the existing literature. Interviews were conducted to collect data on participant demographics, waste disposal activities (including waste collection, waste separation, waste reduction, and waste recycle). Data collection was conducted with a sample size of 15 participants, who were selected based on their availability and preference for convenient locations. The duration of each interview ranged from 60 to 90 min. The researchers conducted interviews in the Thai language and subsequently translated them into the English language. The identical participants were questioned again in three successive rounds: January 2024, April 2024, and July 2024. The interviews are shown in Table 2.

**Table 2:** Questions for interview.

Issue	Interview questions
Participants’ information and motivation	1. Kindly provide a detailed account of your personal and professional history
	2. Please describe your role in Wat Chak Daeng’s waste management.
	3. What is your motivation for engaging in this activity?
	4. What is the current state of waste management at Wat Chak Daeng?
Plastic waste disposal	5. How do you reuse waste in Wat Chak Daeng?
	6. How do you reduce waste in Wat Chak Daeng?
	7. How do you recycle waste in Wat Chak Daeng?
Impact and challenge	8. How does this plastic waste management project affect the environment and local communities?
	9. What are the challenges associated with controlling plastic waste at Wat Chak Daeng?
Eco-Sattva	10. How does the Buddhist teaching of Eco-Sattva influence Wat Chak Daeng’s approach to waste plastic management?
	11. In the context of plastic garbage management, how are spiritual and religious values translated into practical actions?

### Case analysis

The data were analyzed using content analysis, explicitly employing the method outlined by Erlingsson and Brysiewicz (2017). The study was conducted on 15 sets of transcriptions from in-depth interviews. Upon meticulous examination of all the transcriptions, another study team member generated the preliminary codebook. Subsequently, based on the interview data, we produced a collection of coded descriptions that encompassed both concepts and analysis. Researchers examined the codebook for coding a typical interview to assess intercoder validity. The NVivo program was utilized to code and analyze the transcribed data. The program contained lexicons, coding tables for analyzing the document’s co-occurrences, query utilities, and networks. Program-assisted analysis of data comprises five sequential steps: (i) preliminary examination of the data; (ii) thorough examination of the information; (iii) assigning codes to quotes; (iv) grouping and classifying the data; and (v) managing the obtained results.

### Case triangulation

In order to verify that the data were triangulated, we ensured that the data sources were thoroughly validated, that the interview transcripts were examined, and that the findings on the phenomenon were compared (Natow, 2020). Analyzing the data was the responsibility of the first author, while the co-authors were responsible for reviewing and modifying the work. In order to improve the reliability and precision of the data, the co-authors carried out a comprehensive evaluation of the findings and re-examined the subject matter, the coding, and the individual quotations. The essential requirements for ensuring the qualitative validity of a thorough triangulation of data are as follows: (i) comprehensive and detailed data; (ii) validation by the participants involved; (iii) utilization of many sources of data; and (iv) comparison and analysis of the researcher’s observations.

## Results

### Community transformation of Wat Chak Daeng

The study’s findings suggest that the temple serves as a prototype for transitory communities that use advanced waste management technology amid a rapidly growing metropolitan environment. Wat Chak Daeng, situated on Bangkok’s outskirts, transforms a rural setting into a more significant urban development. While this process offers chances to adopt sustainable, community-driven solutions, it also brings out fresh issues in environmental sustainability and waste management. The inquiry examines how these temples incorporate Buddhist precepts into their waste management activities, demonstrating how transitional societies may effectively adapt to change with elegance and unity.

Wat Chak Daeng has introduced a range of measures to encourage sustainable waste management in the context of urbanization. For example, the place of worship follows a strict waste separation system that efficiently applies the 3R principles—Reduce, Re-use, and Recycle—by separating organic and non-organic garbage. These actions are a direct response to the growth of cities and a clear display of a solid commitment to Buddhist principles, such as metta (compassion) and Sati (awareness), specifically regarding the environment. Wat Chak Daeng effectively addresses the waste problem and successfully implements spiritual ideas in environmental management.

This study also reveals the significance of community involvement in refuse management at Wat Chak Daeng. This temple is actively conducting workshops and training sessions for community members by providing environmental awareness education and refuse management techniques. This program not only enhances practical skills but also reinforces Buddhist values that are associated with environmental responsibility and concern. The community’s active participation in this waste management program is crucial for fostering a shared feeling of duty and ownership for ecological safety and facilitating positive transformations within the community. This query poses a problem when implementing Eco-Sattva teachings and trash management.

### Plastic waste management

At Wat Chak Daeng, waste management is a voluntary endeavor that involves the community, community leaders, and monks. This was verified during an interview with the participant, Monk 3, who stated, “I was responsible for the recycling of waste into monk robes.” In addition, the community leader released a statement confirming his involvement in garbage management at the temple. Community leader 2 claimed, “I coordinated the aggregation of plastic refuse and categorized it.” Community volunteer 2, expressed, “I aided in the gathering and handling of plastic waste at the temple.” Subsequently, the researcher inquired, “What is your motivation for participating in this activity?” The participant responded similarly, citing their conviction in the Eco-Sattva teachings. Monk 2’s response: “I am of the opinion that every small action we can take to safeguard the environment is a manifestation of compassion, a virtue that is consistently emphasized in Buddhist teachings and is also the foundation of the Eco-Sattva teaching.” Community leader 1 stated, “I am engaged in this waste management activity because I am concerned about the future of my children and want to guarantee that they have a healthy environment.” The response of this community leader also demonstrates the application of the eco-sattva teachings, which underscore the significance of preserving the environment for all of God’s creatures, both in the present and the future.

The interview results also indicate that the 3R Principle (Reduce, Reuse, and Recycle) is Wat Chak Daeng’s primary pillar of refuse management. This principle is also by the teachings of Eco-Sattva. For instance, the responses of the three monks, Monk 4, Monk 5, and Monk 6. Monk 4 pointed out that “we manage waste using the 3R principle, the real practice is to use refillable water bottles, thereby reducing plastic bottles,” and Monk 5 stated, “we encourage people to use shopping bags made of cloth or paper, thereby reducing plastic waste.” Monk 6 also said, “we appeal to people when they go to the temple to bring their drinking bottles and non-disposable bags.” The three priests’ response demonstrates the concept of reducing plastic waste management by suppressing or reducing the use of disposable plastic materials. This concept is in complete alignment with the teachings of eco-sattva, which emphasize the importance of living a simple existence and developing a love for the environment.

Furthermore, the Eco-Sattva teachings also dominate plastic waste management activities at Wat Chak Daeng in the context of “Reuse.” The Eco-Sattva teachings that monks adhere to and believe in incentivize them to utilize items that are no longer in use, as evidenced by interviews with monks who initiated this waste management. Similarly, in the context of “Recycle,” the three pillars of waste management are delineated in the following manner.

#### Waste reduce

The Eco-Sattva philosophy, rooted in Buddhist precepts and environmental consciousness, is exemplified by the “Waste Reduce” method employed at Wat Chak Daeng for plastic trash management. Eco-Sattva integrates Buddhist teachings, such as awareness and compassion for living beings, with sustainable environmental management practices. The “reduce” process is initiated through education expenditures to enhance the public’s understanding of this issue. The educational program is conducted in the classroom, with the participation of all students. The user is also actively implementing this concept by utilizing the minimum and maximum buttons to indicate the direction of travel or arrival at the site. This rule is mandatory in the school and is strictly enforced by the school’s administrators. Results of the survey with the assistance of Monk 7 and Monk 8, please elaborate on this topic.

The two priests provided mutually supportive responses when the researcher enquired: “How do you reduce waste?”

*“At Wat Chak Daeng, we implement direct and uncomplicated approaches to reduce waste.” Our primary goal is to reduce the use of disposable plastic. We respectfully ask all community members to bring reusable fabric shopping bags and avoid using plastic bags throughout their purchases. Additionally, we minimize the acquisition of disposable products. We also encourage individuals to purchase only genuinely essential items. (Monk 7)*

The researcher then enquired, “In what ways does the Buddhist teaching of Eco-Sattva influence Wat Chak Daeng’s approach to plastic waste management?”

*“We are instructed to live modestly and contribute to preserving the natural order.” We endeavor to mitigate the environmental burden and demonstrate greater benevolence toward the environment by decreasing plastic pollution. The principle of Eco-Sattva can be achieved in ordinary life by implementing small actions, such as avoiding single-use plastic materials. (Monk 8)*

The teachings and principles of eco-sattva are reflected in waste management activities, which reduce the volume of plastic refuse. The genuine practice of this temple is to encourage and request that the public refrain from using disposable materials. The temple management provides cloth bags to visitors who bring plastic or packaging, allowing them to be reused. This practice is consistent with the Buddhist principles of Ahimsa (non-violence) and environmental responsibility. This policy is a practical example for the community regarding ecological care practices in daily activities and reduces the quantity of plastic waste produced. The practice demonstrates that Wat Chak Daeng selects commodities with plastic-free packaging when procuring them. This indicates the Eco-Sattva principle that the administration of Wat Chak Daeng has implemented.

The Eco-Sattva teachings prioritize the Interbeing principle, highlighting all entities’ interconnection. This concept is evident in the fact that every activity has a consequence on the entire ecosystem. The teaching of effective plastic waste management is demonstrated at Wat Chak Daeng through its comprehensive approach to reducing waste. The principle of Interbeing asserts that every choice made in waste management, such as opting for environmentally sustainable alternatives and minimizing plastic usage, has a dual effect: it influences the immediate surroundings. Also, it contributes to the overall well-being of the Earth. The practice at this temple illustrates that fundamental actions can yield a substantial influence by understanding and using this idea. This aligns with the Eco-Sattva teachings, which advocate for the consciousness and accountability of every human toward our environment.

#### Waste reuse

Wat Chak Daeng’s waste utilization program is founded on principles of environmental responsibility, specifically focusing on the “Waste Reuse” stage. Officials at the temple efficiently carry out this directive by discovering innovative ways to repurpose plastic waste. Repurposing plastic waste can efficiently manage resources and showcase resilience for the environment. The results of interviews conducted with Monk 1 priests corroborate these findings.

When researchers enquired, “How do you reuse waste?”

*“At Wat Chak Daeng, we actively participate in the innovative repurposing of waste materials. As an example, we convert plastic bottles into functional items, like as flower pots. Our methodology is simple: we first slice the bottles, adorn them, and subsequently utilize them for cultivating herbs or decorative plants within the temple premises. This technique makes a substantial contribution to improving our environment and reducing trash” (Monk 1)*

*“Waste sorting process Sorting is one of the first mechanisms of waste management. The purpose is to separate waste that can be reused before disposing of waste that cannot be used. If a large amount of waste can be separated for reuse, it can help reduce the amount of waste that needs to be disposed of.” (Community leader 1)*

*“In the first stage of sorting out the garbage, if we find any object that can be reused, we will keep it to be distributed to people who need them. Objects like plastic and glass bottles are reused for containing liquids, such as liquid soap, liquid fertilizer, and so on. Only then will we proceed to the next step of recycling.” (Community leader 2)*

Wat Chak Daeng applies the philosophy of Eco-Sattva by doing various activities to reuse plastic garbage. The main goal is to repurpose used plastic by transforming it into new products, such as plants made from recycled plastic bottles. This approach not only reduces plastic pollution but also maximizes the use of materials that are still usable. This technique educates the community about the importance of improving the worth of things considered worthless, which aligns with Buddhist teachings on Ahimsa (non-violence) toward the environment. The temple administrator conducted a workshop and training session for the community to repurpose this plastic refuse. The interview results corroborate this discovery, as the researchers enquired, “How does the Eco-Sattva concept influence the practice of waste reuse?”

*“The concept of Eco-Sattva significantly influences our approach to the reuse of plastic waste.” The Eco-Sattva principle teaches us that all things are valuable. Eco-Sattva also instills in us the importance of preserving the environment and fostering a passion for it. In addition to reducing the quantity of waste sent to a landfill, the principle of environmental awareness and care is also achieved through reusing waste. (Monk 2)*

#### Waste recycle

In the administration of plastic waste at Wat Chak Daeng, the “Waste Recycle” stage demonstrates implementing the Eco-Sattva principle through sustainable recycling practices. The actual practice that is implemented is recycling plastic refuse into a monk at Wat Chak Daeng. This practice incorporates spiritual values into the recycling process and focuses on recycled technical aspects. The principles of metta (affection) and Ahimsa (without violence) are among them, and they are implemented by considering each component of the plastic debris as a potential resource that can be exploited. When the researcher enquires, “What is your approach to waste recycling?”

*“In Wat Chak Daeng, we are dedicated to the recycling of waste, particularly plastic, as it is one of the most challenging types of waste and has a detrimental effect on the environment.” Our primary initiative involves recycling plastic waste into monks. We first sanitize the plastic the community has collected, then chop and process it into plastic fibers. The sphere is transformed into a thread once the plastic fibers are prepared. These plastic threads are subsequently woven into a durable and supple cloth. The primary material employed in producing a monastic robe is this fabric. The robe that has been made is not only pleasant to wear, but it also carries a profound symbolic significance. This is because it is the outcome of the direct implementation of Buddhist teachings regarding the simplicity and sustainability of the environment and the efforts to protect nature (Monk 7).*

By the Eco-Sattva principle, recycling activities are implemented at Wat Chak Daeng. This temple implements a straightforward recycling process that converts plastic refuse into new raw materials, specifically the yarn that serves as the monk’s raw material. Wat Chak Daeng has implemented a sustainable and efficient recycling system using environmentally friendly methods and basic technology. This action not only helps reduce the amount of plastic pollution but also promotes the responsible reuse of resources. The researcher enquired about potential challenges in implementing the Eco-Sattva teachings in this plastic waste management activity.

*“Certainly, there are obstacles, particularly when it comes to altering one’s ingrained habits. Initially, the majority of the community is not accustomed to the sorting of refuse or the reduction of plastic usage. However, the change is initiated through sustainable education and the example of the monks. Creating consciousness is one of the main challenges. Communities have the ability to exert a substantial impact on the environment through the implementation of small-scale actions, such as utilizing their own reusable shopping bags or opting for products that have a lesser negative impact on the environment. At first, it is just an idea, but then it becomes concrete actions that are carried out every day.” (Monk 7)*

The pre-dhamma and pre-somchit monks responded favorably when researchers enquired about the impact of plastic waste management on the local community and the adjacent environment. This waste management activity reduces the volume of refuse and contains a significant component: the implementation of Buddhist Eco-Sattva.

*“This waste management has a significant impact on the local community and the surrounding environment. It is evident that there has been a significant decrease in the amount of plastic waste in our surroundings. Plastic waste, once a major environmental concern, has now become a valuable resource thanks to recycling and reusing. In addition, the air and water in the area surrounding the temple have seen a noticeable improvement due to the enhanced management of a substantial amount of plastic waste that was previously discarded or incinerated.” (Monk 1)*

*“Waste management has a highly beneficial effect on local communities. The community is becoming increasingly aware of the significance of environmental protection. Numerous members of the community who were previously indifferent to environmental concerns are now becoming more actively engaged in a variety of refuse management initiatives. Furthermore, this activity create novel economic prospects, such as the sale of recycled products or the use of compost from organic waste in agriculture.” (Monk 2)*

## Discussion

The findings of the In-Depth Interview with 15 Participants address research questions, more specifically, the application of Eco-Sattva teachings in the management of plastic waste in Thailand and their impact on Buddhist communities and local communities, particularly at Wat Chak Daeng. In general, the interview results suggest that the teachings of Eco-Sattva regarding plastic refuse management are highly beneficial in terms of their practical application and implementation. These findings are based on previous research that underscores the potential of spiritual values and religious teachings to influence behavioral changes in response to environmental concerns. For example, a study by Norberg-Hodge et al. (2001) discovered that the community in Ladakh, India, could seamlessly incorporate ecological principles into their daily lives, including waste management. Similarly, Swearer (2010) found that the Buddhist movement referred to as “Engaged Buddhism” supported the active participation of its followers in environmental conservation, which closely mirrored the approach adopted at Wat Chak Daeng.

However, despite the similarities, various studies have highlighted the difficulties in utilizing religious teachings for environmental objectives. As an illustration, a survey conducted by Gombrich (2009) in Sri Lanka uncovered the discrepancy between the ecological preservation ideals promoted by Buddhist teachings and the actual practices on the ground. Financial limitations and deeply rooted cultural customs hinder the achievement of these goals. On the other hand, the findings from Wat Chak Daeng demonstrate that Eco-Sattva teachings are not just discussed in theory but are also put into action through practical, sustainable waste management projects. In Thailand, Walker (2014) conducted research highlighting the challenges faced in implementing Buddhist teachings, emphasizing the importance of environmental preservation. Economic barriers and inadequate infrastructure often hinder the actualization of these teachings.

Furthermore, further research suggests that the specific circumstances and guidance of the local community significantly impact the incorporation of religious teachings into environmental management. For example, Taylor researched Myanmar and found that an influential spiritual leader and influencer was crucial in inspiring the community to engage in environmental preservation initiatives. The director of the monastery and the monks have a vital role in encouraging the community to adopt the principles of Eco-Sattva in managing plastic waste, as demonstrated by Wat Chak Daeng. In Cambodia, a study by King (2017) revealed that the community’s backing for environmental initiatives spearheaded by the temple is heavily influenced by the tangible actions arising from religious teachings. Wat Chak Daeng is successful in this regard. There is also evidence that when religious teachings are internalized deeply, they can generate significant and sustainable changes in behavior, in contrast to research that demonstrates challenges. For instance, Tucker and Williams (1997) conducted research in Tibet that demonstrated that Buddhist teachings regarding the sanctity of nature were instrumental in mitigating environmental degradation in the region. This is consistent with the discoveries at Wat Chak Daeng, where the teachings of Eco-Sattva are not only moral guidelines but are also implemented in daily activities, including environmental education, recycling, and plastic reduction. This concept is also substantiated by research conducted by Schmithausen (1997), which demonstrates that Buddhist teachings can serve as a robust foundation for sustainable environmental initiatives when coupled with effective leadership and community involvement.

### Recommendations

(1) Thai government agencies should promote eco-sattva education in schools, other temples, and public spaces by revising educational standards to include environmental sustainability, Buddhist teachings on nature, and the waste management model from Wat Chak Daeng. Thai government agencies should invite Buddhist monks from Wat Chak Daeng to teach the waste management and recycling process to schools, other temples, and public spaces. At the same time, the Thai government should enforce very strict waste management laws like Singapore.

(2) Religious institutions: the abbot of other temples in Thailand should send the monks in their temple to go to learn and copy the successful waste management and recycling process model from Wat Chak Daeng in order to apply it in their temples.

(3) Local communities. Community leaders should be trained in the concept of eco-sattva, encouraging sustainable consumption and a waste management model from Wat Chak Daeng. Community leaders should develop community waste management programs by following the waste management model from Wat Chak Daeng. They also should set campaign “plastic-free days” in order to reduce plastic waste in their community and also campaign calling for donations of plastic bottles to Wat Chak Daeng.

### Limitation of the study and suggestions for future research

This study has several limitations that may influence the interpretation and generalizability of its findings.

First, the research was limited to only one case study as the focus of the research. While this provided valuable insights into the waste management process of Wat Chak Daeng, the findings may not be applicable to other locations with different attributes. The findings may not be generalizable to eco-sattva movements in a broader context. Including a more diverse sample in future research could help determine the extent to which the results apply to other regions, enhancing the applicability of the study’s generalization.

Second, the data was collected merely through observation and interviews on brief visits. Incorporation of ethnographic participant observation can provide a broader and nuanced understanding of the process.

Third, the study was designed in a qualitative research approach neglecting incorporation of a quantitative aspect. Utilizing a mixed method would enhance the validity of the research.

Recognizing these limitations provides a basis for future research to build upon and expand the scope of this study, thereby contributing to a more comprehensive understanding of the application of the Eco-Sattva concept into environmental activism in a broader context.

## Data Availability

The raw data supporting the conclusions of this article will be made available by the authors, without undue reservation.
